# Comparison of Short-Term Outcomes and Survivorship of Three Modular Dual Mobility Implants in Primary Total Hip Surgery

**DOI:** 10.3390/jcm14196977

**Published:** 2025-10-01

**Authors:** Mitchell Kennedy, Braden Terner, Chukwuweike Gwam, Ran Schwarzkopf

**Affiliations:** Department of Orthopedic Surgery, NYU Langone Health, New York, NY 10003, USA; braden.terner@nyulangone.org (B.T.); chukwuweike.gwam@nyulangone.org (C.G.); ran.schwarzkopf@nyulangone.org (R.S.)

**Keywords:** arthroplasty, hip replacement hip, hip prosthesis, survivorship analysis, treatment outcomes, patient reported outcome measures, modular dual mobility

## Abstract

**Background**: Total hip arthroplasty (THA) is a common procedure, yet instability and dislocation remain leading causes of revision. Dual mobility (DM) acetabular constructs improve stability, but comparative data across modular DM systems are limited. This study compared the safety and efficacy of three modular DM implants in primary THA, focusing on acetabular revision and functional recovery. **Methods**: We retrospectively reviewed 963 primary THAs performed from 2016–2024 using three modular DM systems. Patients with revision or bilateral THA, age < 18, or <2 years of follow-up were excluded. Outcomes included acetabular revision, 90-day readmission, and Hip Disability and Osteoarthritis Outcome Score for Joint Replacement (HOOS, JR). Kaplan–Meier analysis estimated 3-year implant survivorship for each implant, and non-inferiority of Implant A was tested against a combined “Dual Mobility Control” cohort (Implants B + C) using a prespecified −10% margin. **Results**: A total of 297 patients met inclusion criteria (142 Implant A, 110 Implant B, 45 Implant C). Revision rates were 4.9% for Implant A, 6.4% for Implant B, and 8.9% for Implant C. HOOS, JR scores improved significantly in all cohorts with comparable 2-year outcomes. Kaplan–Meier analysis showed 3-year survivorship of 98.3% for Implant A, 98.4% for Implant B, and 96.9% for Implant C (log-rank *p* = 0.053). The Dual Mobility Control cohort survivorship was 98.0%, and the difference between Implant A and controls (95% CI: −2.19% to 2.69%) met the non-inferiority margin (log-rank *p* = 0.796). **Conclusions**: Implant A demonstrated non-inferior 3-year survivorship and comparable short-term patient-reported outcomes relative to two other modular DM implants. Larger, multicenter studies with longer follow-up are warranted to confirm these findings.

## 1. Introduction

Total hip arthroplasty (THA) is one of the most common surgical procedures, with a lifetime risk of 8–16% in developed countries [1,2,3,4]. Demand continues to rise due to an aging population and broader surgical indications, with projections estimating a 176% increase in annual THA volume by 2040 and up to 469% by 2060 [2,5,6].

Mechanical complications—particularly instability and dislocation—remain leading causes of THA revision [7,8,9,10]. Reported dislocation rates range from 2–3% within two years and approach 10% with longer-term follow-up [8,10,11,12]. Instability and dislocation are major contributors to patient morbidity and increased healthcare costs, often leading to pain, loss of function, and the need for additional surgical intervention—all of which are factors associated with longer hospital stays and higher readmission rates [13].

The dual mobility (DM) construct was developed in the 1970s to enhance stability without sacrificing range of motion, mitigating the risk of dislocation and instability [14,15,16,17]. Recent studies demonstrate favorable outcomes with DM implants. A 2022 systematic review found DM designs were associated with lower rates of all-cause revision, fracture-related revision, and dislocation compared to large-head implants [17]. Other studies have similarly reported higher patient satisfaction at 3, 6, and 12 months in DM recipients [18]. Other studies confirm reduced dislocation risk in high-risk populations, including patients with neuromuscular disease or abductor deficiency, and show these low dislocation rates are reproducible even with variable surgeon experience [19,20].

However, direct comparisons of different modular DM designs within the same patient population remain scarce. Most studies are limited by single-surgeon or single-manufacturer cohorts, which restrict generalizability. While others have compared anatomic and modular DM systems from the same manufacturer and reported favorable outcomes, the single-surgeon, single-vendor design limits broader applicability [21]. Likewise, a recent monocentric series demonstrated excellent survival of second-generation uncemented DM cups compared to first-generation cups, but both implants were from the same manufacturer [22]. Studies have compared ADM, MDM, and fixed-bearing constructs but reported HOOS, JR outcomes only for 1 year, limiting longer-term PROM comparison [23].

Variations in implant geometry, liner retention mechanisms, and articulation clearances may affect both implant stability and wear performance [24]. Understanding their relative performance is essential for surgical planning and optimizing patient outcomes. The purpose of the present study is to perform this comparison in the case of three modular DM implants, one of which is a recent addition to the clinic and two that have been in longer clinical use. The performance metrics determined included patient outcomes at 2 follow-up and estimated 3-year survivorship.

## 2. Methods

### 2.1. Study Design

**Figure 1:**
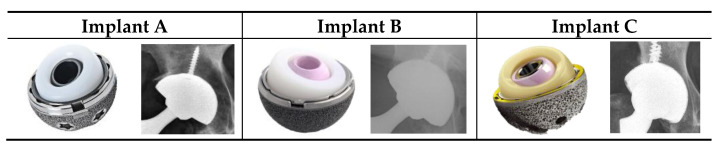
Implant images and x-rays of Implant A, Implant B, and Implant C.

Following institutional review board (IRB) approval, we conducted a retrospective review of 963 primary THAs performed between June 2016 and January 2024 using one of three modular dual mobility implants: Implant A (Smith & Nephew OR3O, Memphis, TN, USA), Implant B (Stryker MDM, Mahwah, NJ, USA), or Implant C (Zimmer G7 DM, Warsaw, IN, USA) (Figure 1). Inclusion criteria were primary THA with one of the above implants. Exclusion criteria included revision THA, bilateral THA, age < 18 years, or follow-up < 2 years. Outcomes and survivorship were analyzed for each implant cohort individually to enable direct comparison. Revision events were defined as procedures involving the acetabular component, including the acetabular liner, cup, or both.

All three implants are designed to enhance stability and range of motion in THA. Implant A features a highly cross-linked ultra-high-molecular-weight polyethylene (HXLPE) insert produced via a proprietary crosslinking process to reduce wear and osteolysis. Its liner is made of diffusion-hardened oxidized zirconium, and a titanium acetabular shell [25]. Implant B utilizes an X3 highly cross-linked polyethylene insert with a cobalt-chromium modular liner and a titanium acetabular shell [26]. Implant C employs a Vivacit-E highly cross-linked polyethylene insert crosslinked via electron beam irradiation, with a cobalt-chromium liner and a titanium acetabular shell [27].

Because Implants B and C share similar cobalt–chromium liner materials, they were combined into a single “Dual Mobility Control” cohort to increase statistical power for non-inferiority comparisons against Implant A, which uses a DH zirconium liner [28,29,30,31]. The material composition and design features of all three implants are summarized in Table 1.

### 2.2. Data Collection and Outcome Measures

Patient data were extracted from the institutional electronic medical record database (Epic Caboodle Version 15; Verona, WI, USA). Collected demographic variables included age at surgery, BMI, sex, race, smoking status, and American Society of Anesthesiologists (ASA) classification.

The primary outcome was all-cause acetabular revision of the implant device. Secondary outcomes included 90-day hospital readmissions and patient-reported outcome measures (PROMs), specifically the Hip Disability and Osteoarthritis Outcome Score for Joint Replacement (HOOS, JR), collected preoperatively and at 6 weeks, 3 months, 1 year, and 2 years postoperatively. Analyses of demographics and clinical outcomes were restricted to patients with at least 2 years of follow-up.

### 2.3. Data Analysis

Descriptive statistics were calculated as means, counts, standard deviations, and ranges. Outcomes and survivorship were analyzed for each implant cohort individually (Implant A, Implant B, Implant C) and for the combined Dual Mobility Control cohort. Baseline characteristics were reported as counts with percentages for categorical variables and as means with ranges and standard deviations for continuous variables. All analyses were conducted using R (version 4.4.2, Vienna, Austria).

To account for potential confounding due to demographic or clinical differences between implant groups, a multivariate Cox proportional hazards regression was performed, including implant type, age, sex, BMI, ASA class, race, and smoking status as covariates. Hazard ratios with 95% confidence intervals were calculated for each covariate to quantify their association with risk of revision to ensure that implant group differences were not explained by baseline population heterogeneity.

For patient-reported outcomes, HOOS, JR scores were compared across the three implant cohorts at each follow-up time point using one-way analysis of variance (ANOVA). When the ANOVA indicated potential differences, Tukey’s honestly significant difference (HSD) post hoc test was performed for pairwise comparisons between implant groups.

A priori power calculations estimated the sample size needed to demonstrate non-inferiority of Implant A compared with other modular DM systems. Based on the Australian Orthopedic Association of Joint Replacement Registry, the expected 3-year survivorship was 97.2% for all dual mobility implants and 99.5% for Implant A (revision rate 0.4%) [32]. Assuming a 10% non-inferiority margin [33], and α = 0.05, a two-sided Fisher’s Exact test indicated that 68 subjects per group would provide > 80% power, while 99 subjects per group would achieve ≥ 99% power.

Three-year implant survivorship was estimated using Kaplan–Meier analysis for the entire cohort, including patients with <2 years of follow-up, and truncated at 1095 days to standardize follow-up. Kaplan–Meier estimates provided the probability of implant survival at each time point for individual implants and the combined control group, with approximate 95% confidence intervals calculated to quantify uncertainty. Non-inferiority of Implant A was formally assessed using the 95% confidence interval for the difference in 3-year KM survivorship versus the combined Dual Mobility Control cohort, with non-inferiority concluded if the lower bound exceeded the prespecified −10% margin. Log-rank tests were used to compare survival curves across the three implants individually and between Implant A and the combined control cohort.

## 3. Results

Of the 297 patients with ≥2 years of follow-up, 142 received Implant A, 110 Implant B, and 45 Implant C. The mean age was 62.7 years (range 22–91) and did not differ significantly between groups (*p* = 0.809). Mean BMI was 29.3 kg/m^2^ and differed across implants (*p* = 0.023), with Implant B patients having a lower BMI (27.8 kg/m^2^). Pairwise comparisons showed BMI was significantly higher in the Implant A group compared to Implant B (*p* = 0.022), with no significant differences between Implant C and the other implants.

Female representation ranged from 51.4% in Implant A to 68.9% in Implant C (*p* = 0.057). Most patients were White, with race differing significantly across groups (*p* = 0.044) due to a higher proportion of Black or African American patients in Implant A (21.8%) versus Implant B (6.4%) and C (11.1%). Pairwise comparisons confirmed significant differences between Implant A and Implant B (*p* = 0.009), but not between Implant A and Implant C or Implant C and Implant B.

Approximately half of patients were never smokers (*p* = 0.289), and 59.3% were ASA class II. Mean follow-up differed statistically (*p* < 0.001), with Implant A at 3.09 years (2.00–4.91), Implant B at 3.83 years (2.01–8.17), and Implant C at 3.38 years (2.01–6.84). Laterality (*p* = 0.860) and fixation method (cemented vs. uncemented, *p* = 0.275) were similar across cohorts. Full descriptive demographics are presented in Table 2.

Seventeen patients (5.7%) were readmitted within 90 days for surgery-related causes, most commonly infection or femur fracture (Table 3). Readmission rates were 4.9% for Implant A, 3.6% for Implant B, and 13.3% for Implant C, with no statistically significant difference across groups (*p* = 0.077). When Implants B and C were combined into a Dual Mobility Control cohort, the 90-day readmission rate was 6.5%, which did not differ significantly from Implant A (*p* = 0.625; OR 0.75, 95% CI 0.24–2.26).

Eighteen patients (6.1%) underwent acetabular revision within three years (Table 3). Revision rates were 4.9% for Implant A, 6.4% for Implant B, and 8.9% for Implant C (*p* = 0.576). The combined Dual Mobility Control cohort had a 3-year revision rate of 7.1%, not significantly different from Implant A (*p* = 0.576; OR 0.64, 95% CI 0.23–1.76). Reasons for revision included dislocation/instability (n = 3), infection (n = 2), periprosthetic fracture (n = 1), and malposition (n = 1) for Implant A; periprosthetic fracture (n = 2), infection (n = 1), aseptic loosening (n = 3), and pseudotumor (n = 1) for Implant B; and periprosthetic fracture (n = 1), infection (n = 1), and aseptic loosening (n = 2) for Implant C.

Patient-reported outcome measures were similar across all three implant cohorts at each follow-up (Table 4). All groups experienced improvements in HOOS, JR scores, and one-way ANOVA with Tukey post hoc comparisons showed no statistically significant differences at any time point, indicating comparable functional outcomes.

When Implants B and C were combined into a Dual Mobility Control cohort, pre- and postoperative HOOS, JR trajectories remained similar, with no significant differences compared to Implant A. Although not statistically significant, Implant A patients had higher mean HOOS, JR scores at preoperative (48.7 vs. 47.1), 3 months (73.6 vs. 64.8), 1 year (79.6 vs. 75.8), and 2 years (78.9 vs. 71.2) relative to the combined DM control group. Additional HOOS, JR details are provided in Table 5.

Kaplan–Meier survival analysis was performed for the full cohort, including patients with <2 years of follow-up, to estimate 3-year implant survivorship. This cohort included 406 Implant A, 429 Implant B, and 128 Implant C cases, with curves truncated at 1095 days to standardize follow-up. Estimated 3-year survivorship was high across all groups: 98.26% for Implant A, 98.36% for Implant B, and 96.86% for Implant C (Table 6), with no significant difference by log-rank test (χ^2^ = 1.3, df = 2, *p* = 0.530).

**Figure 2:**
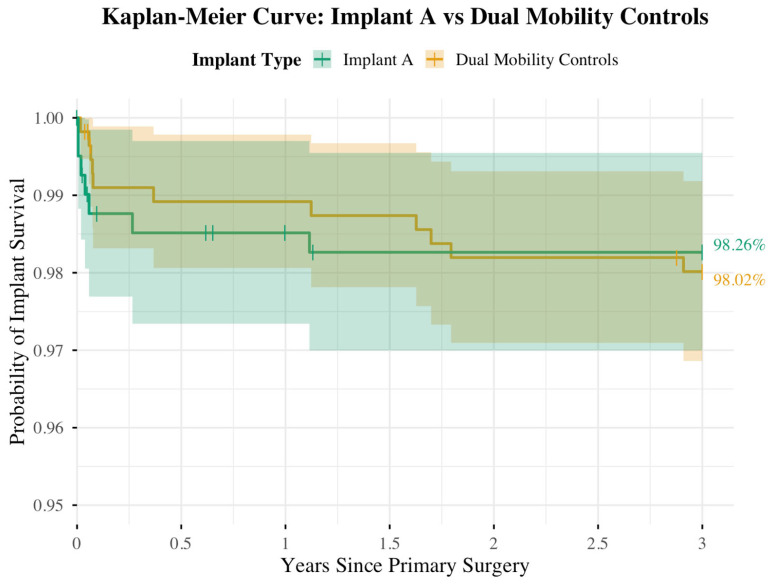
Kaplan–Meier implant survivorship over three years: Implant A Cohort versus Dual Mobility Controls Cohort.

For the primary non-inferiority analysis, the combined Dual Mobility Control group had a 3-year survivorship of 98.02% (Figure 2). Comparing Implant A (98.26%; 95% CI: 96.97–99.55%) to the control cohort (98.02%; 95% CI: 96.86–99.18%), the 95% CI for the difference in survivorship was −2.19% to 2.69%, well within the prespecified −10% non-inferiority margin, demonstrating non-inferiority of Implant A (Table 7).

The survival curves remained flat over the 3-year period, with no significant difference in implant survivorship between Implant A and the Dual Mobility Control cohort (log-rank χ^2^ = 0.10, df = 1, *p* = 0.796). The risk table summarizes patients at risk, events, and censored cases at 6-month intervals (0, 0.5, 1, 1.5, 2, 2.5, and 3 years) (Table 8). Combining Implants B and C into the Dual Mobility Control group yielded a sample size that exceeded both the >80% and ≥99% power thresholds to demonstrate non-inferiority within the prespecified margin.

Cox proportional hazards modeling including implant type, age, sex, BMI, ASA class, race, and smoking status revealed no statistically significant predictors of revision within three years (overall model likelihood ratio test: *p* = 0.801). None of the demographic or clinical covariates (age, sex, BMI, ASA, race, or smoking status) demonstrated a significant independent effect on revision risk.

## 4. Discussion

Total hip arthroplasty (THA) is one of the most frequently performed procedures in the United States, with demand projected to continue rising [1,2,3,4,5,6]. Periprosthetic dislocation remains a serious postoperative complication, contributing to increased morbidity, higher costs, and decreased patient satisfaction [34,35,36,37,38]. Dual mobility constructs have been developed to reduce dislocation risk by increasing impingement-free range of motion and jump distance [39,40], making them particularly advantageous for high-risk patients, including those with neuromuscular disorders, poor abductor function, seizure disorders, or undergoing revision surgery. In this single-institution retrospective study, we evaluated short-term outcomes following primary THA using three dual mobility constructs (Implants A, B, and C). We observed similar short-term outcomes between groups, with 90-day all-cause readmission rates of 6.4% for the combined Dual Mobility Control group (Implants B and C) and 4.9% for Implant A at three years.

The comparable outcomes and survivorship of Implant A relative to the Dual Mobility Control cohort may be attributed to its design and material characteristics. Oxidized zirconium is associated with lower metal ion release compared with cobalt-chrome, potentially reducing the risk of CoCr related adverse events [41,42,43]. Additionally, oxidized zirconium is associated with lower metal ion release compared with cobalt-chrome, potentially reducing the risk of metal-related adverse events [44]. While direct comparisons between oxidized zirconium–HXLPE and cobalt chromium–HXLPE have shown no statistically significant differences in wear or survivorship [42,45,46], our findings suggest that the Implant A design utilizing an oxidized zirconium liner may confer at least comparable, and possibly superior, clinical outcomes.

In line with our results which demonstrated no statistically significant differences in HOOS, JR scores between implant groups, the higher mean scores observed for Implant A—ranging from 7 to 9 points at key postoperative intervals (e.g., 3 months and 2 years)—are likely clinically meaningful. Prior anchor- and distribution-based studies have established minimal clinically important difference (MCID) thresholds for HOOS, JR of 7.8–9 points at 1 year following primary THA [47,48], suggesting that the observed differences may represent perceptible improvements in hip function and symptoms. Taken together, these findings indicate that while all implant groups experienced substantial postoperative improvement, Implant A may provide at least equivalent, and potentially superior, short-term patient-reported outcomes compared with other modular dual mobility systems.

Our study demonstrated that Kaplan–Meier estimated 3-year survivorship was 98.26% for implant A and 98.02% for the DM control groups. To place our results in context, we compared them with prior studies of modular dual mobility (DM) implants in primary THA. Studies were selected if they included primary THA with modular DM constructs, reported patient age distribution, fixation method, and had at least two years of follow-up. Ruusiala et al. reviewed 101 patients (mean age 66 years, range 18–90) and reported 2-year survivorship of 97% in primary THA with a dislocation rate of 1.4% [49]. These outcomes are consistent with the excellent 3-year survivorship observed in our cohort (98.26% for Implant A and 98.02% for the DM controls). Khatod et al. analyzed 107,528 primary THAs (mean age 68 years, range 18–95) with a mean follow-up of 6 years and demonstrated that DM implants reduced revision and dislocation risk compared to conventional implants [50]. Schaffer et al. examined 61 patients (mean age 61 years, range 30–85) with a mean follow-up of 2.4 years, showing low revision rates and favorable patient-reported outcomes, consistent with the present study [51]. Collectively, these findings support the conclusion that modular DM implants provide reliable short-term survivorship and functional improvement across different populations and implant systems.

Using the prespecified 10% non-inferiority margin, the 3-year Kaplan–Meier survivorship analysis demonstrates non-inferiority of implant A relative to the group of the two control DM implants. Additionally, log-rank analysis showed that the difference in implant survivorship between the two groups was not significant (*p* = 0.796). These findings, which are consistent with the observed Kaplan–Meier curves and low revision rates, indicate that, in the short term (3 years), the survivorship of the relatively new DM construct (Implant A) is comparable to that of the more established constructs (Implants B and C).

This study has several limitations. First, it was conducted at a single institution, which may limit generalizability. Follow-up was relatively short, and longer-term studies are needed to assess implant performance over the lifespan of an aging population. The combination of Implants B and C into a single Dual Mobility Control cohort may have masked potential differences between these systems, which should be considered when interpreting comparative outcomes. Important patient outcomes, such as serum metal ion levels and characterization of metal ion release, were not obtained for this cohort and could have provided insight into the real-world corrosion resistance of dual mobility constructs. Although the a priori power analysis indicated the sample size exceeded thresholds to demonstrate non-inferiority within a 10% margin for 3-year survivorship, the limited number of revision events and follow-up preclude definitive conclusions regarding long-term equivalence. Despite the observed 3-year revision rate for Implant A being higher than initially assumed, the study remained adequately powered to assess non-inferiority, though this difference should be considered when interpreting absolute revision rates. Finally, potential confounders such as individual surgeon technique, surgical approach, or specific polyethylene liner characteristics were not consistently recorded and may have influenced implant survival outcomes.

## 5. Conclusions

With regard to patient outcomes at 3 years, such as all-cause acetabular component revision rate, 90-day readmission rate, HOOS, JR score and the estimated 3-year Kaplan–Meier survivorship, we found that a recently introduced dual mobility implant is comparable to that of a set of 2 well-established dual mobility implants in spite of differences in design features between them, such as UHMWPE insert locking geometry and femoral head material. While this finding suggests a widening of the choice of DM implant designs that THA surgeons should consider for a given case, longer-term, multicenter studies are needed to test its validity.

## Figures and Tables

**Table 1:** Dual Mobility Implant Characteristics.

Component	Implant A	Implant B	Implant C
Insert	Highly Cross-Linked UHMWPE (proprietary crosslinking process)	X3 Highly Cross-Linked UHMWPE (gamma irradiation + thermal treatment)	Vivacit-E Highly Cross-Linked UHMWPE (electron beam irradiation with vitamin E stabilization)
Modular Liner	DH Oxidized Zirconium	Cobalt–Chromium	Cobalt–Chromium
Acetabular Shell	Titanium alloy shell	Titanium alloy shell	Titanium alloy shell

DH, diffusion hardened; UHMWPE, ultra-high-molecular-weight polyethylene; XLPE, cross-linked polyethylene.

**Table 2:** Patient Demographic Data.

	Implant A (n = 142)	Implant B (n = 110)	Implant C (n = 45)	Total (n = 297)	
**Age** (years), mean [range]	62.3 [26–87]	63.2 [22–84]	63.1 [25–91]	62.7 [22–91]	*p* = 0.809
**BMI** (kg/m^2^), mean [range]	29.9 [19.2–51.1]	27.8 [15.4–44.6]	29.9 [19.9–46.6]	29.3 [15.4–51.1]	***p* = 0.023**
**Sex,** n (%)					*p* = 0.057
Female	73 (51.4)	69 (62.7)	31 (68.9)	173 (58.2)	
Male	69 (48.6)	41 (37.3)	14 (31.1)	124 (41.8)	
**Race,** n (%)					***p* = 0.044**
White	85 (59.9)	79 (71.8)	32 (71.1)	196 (66.0)	
Black or African American	31 (21.8)	7 (6.4)	5 (11.1)	43 (14.5)	
Hispanic or Latino or Spanish	5 (3.5)	4 (3.6)	2 (4.4)	11 (3.7)	
Other	21 (14.8)	20 (18.2)	6 (13.3)	47 (15.8)	
**Smoking Status,** n (%)					*p* = 0.289
Never	64 (45.0)	61 (55.5)	27 (60.0)	151 (51.2)	
Former	61 (43.0)	41 (37.3)	15 (33.3)	117 (39.9)	
Current	17 (12.0)	8 (7.3)	3 (6.7)	28 (9.4)	
**ASA Score**, n (%)					*p* = 0.061
I	15 (10.6)	10 (9.1)	5 (11.1)	30 (10.1)	
II	88 (62.0)	70 (63.6)	18 (40)	176 (59.3)	
III	36 (25.4)	30 (27.3)	20 (44.4)	86 (29.0)	
IV	3 (2.1)	0 (0.0)	2 (4.4)	5 (1.7)	
**Follow-Up** (years), mean [range]	3.1 [2.0–4.9]	3.8 [2.0–8.2]	3.4 [2.0–6.8]	3.4 [2–8.2]	***p* < 0.001**
**Laterality** Right Left	79 63	55 55	27 18	161 136	*p* = 0.860
**Fixation Method** Cemented Uncemented	16 126	3 107	9 36	28 269	*p* = 0.275

BMI, body mass index; kg, kilograms; m, meters; ASA, American Society of Anesthesiologists.

**Table 3:** Two-Year Clinical Outcomes.

Outcome	Implant A	Implant B	Implant C	Total
All-cause Acetabular Component Revision, n (%)	7 (4.9)	7 (6.4)	4 (8.9)	18 (6.1)
Dislocation/Instability	3	0	0	3
Infection	2	1	1	4
PPF	1	2	1	4
Malposition	1	0	0	1
Aseptic Loosening	0	3	2	5
Pseudotumor	0	1	0	1
90-day readmissions, n (%)	7 (4.9)	4 (3.6)	6 (13.3)	17 (5.7)
Infection	2	0	1	3
PPF	1	0	0	1
Mechanical Loosening	0	0	2	2
DVT	0	0	2	2
Femur Fracture	1	2	0	3
Anesthesia of Skin	1	0	0	1
Primary Osteoarthritis	0	2	0	2
Other *	2	0	1	3

PPF, Periprosthetic fracture; DVT, Deep Vein Thrombosis. * Other: Iron Deficiency Anemia, Spastic Cerebral Palsy, Encephalopathy.

**Table 4:** Hip Dysfunction and Osteoarthritis Outcome, Joint Replacement (HOOS, JR) Scores Across Individual Implants.

	Implant A	Implant B	Implant C	*p*-Value
Preoperative	48.7 (16.1) [72]	47.9 (14.4) [42]	44.4 (15.0) [12]	0.676
6 weeks	63.4 (14.9) [73]	64.8 (13.4) [41]	67.0 (14.2) [13]	0.667
3 months	73.6 (18.5) [71]	62.1 (31.9) [31]	71.7 (11.4) [12]	0.064
1 year	79.6 (18.2) [50]	78.8 (15.7) [29]	69.6 (14.3) [14]	0.147
2 years	78.9 (21.6) [51]	67.4 (26.2) [19]	80.3 (16.6) [8]	0.142

Results given as mean (standard deviation) [number of patients].

**Table 5:** Hip Dysfunction and Osteoarthritis Outcome, Joint Replacement (HOOS, JR) Scores Across Implant A and DM Control.

	Implant A	Dual Mobility Control	*p*-Value
Preoperative	48.7 (16.1) [72]	47.1 (14.5) [54]	0.578
6 weeks	63.4 (14.9) [73]	65.4 (13.5) [54]	0.441
3 months	73.6 (18.5) [71]	64.8 (27.9) [43]	0.072
1 year	79.6 (18.2) [50]	75.8 (15.7) [43]	0.276
2 years	78.9 (21.6) [51]	71.2 (24.0) [27]	0.167

Results given as mean (standard deviation) [number of patients].

**Table 6:** Implant Specific Three-year Kaplan–Meier Implant Survivorship Estimates.

Implant Group	3-Year KM Survivorship (%)	95% CI for Survivorship (%)
Implant A	98.26	96.97–99.55
Implant B	98.36	97.16–99.57
Implant C	96.86	93.91–99.57

KM, Kaplan–Meier; CI, Confidence Interval.

**Table 7:** Combined Three-year Kaplan–Meier Implant Survivorship Estimates.

Implant Group	3-Year KM Survivorship (%)	95% CI for Survivorship (%)	95% CI for Difference (%)
Implant A	98.26	96.97–99.55	−2.19–2.69
DM Controls	98.02	96.86–99.18	

KM, Kaplan–Meier; CI, Confidence Interval.

**Table 8:** Number of Patients at Risk, Over Three Years.

Implant Group	0 Yrs	0.5 Yrs	1 Yrs	1.5 Yrs	2 Yrs	2.5 Yrs	3 Yrs
Implant A							
n, at risk	406	396	393	391	391	391	391
n, events	0	6	0	1	0	0	0
n, censored	0	4	3	1	0	0	0
DM Controls							
n, at risk	557	548	548	547	544	544	542
n, events	0	6	0	1	3	0	1
n, censored	0	3	0	0	0	0	1

Yrs, Years.

## Data Availability

All data is available to be reviewed upon request.
